# Identification of Potential Prognostic Biomarkers for Breast Cancer Based on lncRNA-TF-Associated ceRNA Network and Functional Module

**DOI:** 10.1155/2020/5257896

**Published:** 2020-07-28

**Authors:** Xinrong Li, Junquan Zhu, Jian Qiu

**Affiliations:** Department of Integrative Medicine & Medical Oncology, Shengzhou People's Hospital (The First Affiliated Hospital of Zhejiang University Shengzhou Branch), 312400, Shengzhou, Zhejiang, China

## Abstract

Breast cancer leads to most of cancer deaths among women worldwide. Systematically analyzing the competing endogenous RNA (ceRNA) network and their functional modules may provide valuable insight into the pathogenesis of breast cancer. In this study, we constructed a lncRNA-TF-associated ceRNA network via combining all the significant lncRNA-TF ceRNA pairs and TF-TF PPI pairs. We computed important topological features of the network, such as degree and average path length. Hub nodes in the lncRNA-TF-associated ceRNA network were extracted to detect differential expression in different subtypes and tumor stages of breast cancer. MCODE was used for identifying the closely connected modules from the ceRNA network. Survival analysis was further used for evaluating whether the modules had prognosis effects on breast cancer. TF motif searching analysis was performed for investigating the binding potentials between lncRNAs and TFs. As a result, a lncRNA-TF-associated ceRNA network in breast cancer was constructed, which had a scale-free property. Hub nodes such as *MDM4*, *ZNF410*, *AC0842-19*, and *CTB-89H12* were differentially expressed between cancer and normal sample in different subtypes and tumor stages. Two closely connected modules were identified to significantly classify patients into a low-risk group and high-risk group with different clinical outcomes. TF motif searching analysis suggested that TFs, such as *NFAT5*, might bind to the promoter and enhancer regions of hub lncRNAs and function in breast cancer biology. The results demonstrated that the synergistic, competitive lncRNA-TF ceRNA network and their functional modules played important roles in the biological processes and molecular functions of breast cancer.

## 1. Introduction

Breast cancer is one of the most common female cancers worldwide, which is also the second leading cause of female cancer death [1]. Adjuvant therapy has been an effective way to improve patient survival and promote the quality of life [2]. However, tumor metastasis and drug resistance are still a concern during breast cancer therapy. Thus, there is an urgent need to identify key biomarkers and uncover potential molecular mechanisms for breast cancer diagnosis and therapy. Many studies have identified some important genes that participated in the occurrence, development, and metastasis of breast cancer. For example, two well-known cancer genes, *BRCA1* and *BRCA2*, were the major genes associated with the genetic etiology of breast cancer. Women with *BRCA1/BRCA2* mutations had very high risk to develop breast cancer [3]. Mutations or variants of other genes such as *TP53*, *ATM*, *BARD1*, *CHECK2*, *FGFR2*, *GSTM1*, and *MAP3K1* have also been reported to increase the risk of breast cancer [4].

Long noncoding RNAs (lncRNAs) are a type of RNA transcript of more than 200 nucleotides, which have been considered effective disease biomarkers in cancers [5]. Abnormal expression of several lncRNAs has been shown to be involved in breast cancer. For example, lncRNA *HOTAIR* was overexpressed and acted as a powerful predictor of metastasis in breast cancer [6]. The depletion of lncRNA *MALAT1* decreased the tumorigenesis and metastasis of breast cancer [7]. lncRNA *AGAP2-AS1* could promote breast cancer cell growth by upregulating the expression of *MyD88* and activating the NF-*κ*B signaling pathway [8]. In addition to these important functions in breast cancer, many recent studies have reported that lncRNAs might interact with mRNAs, competitively bind to their common microRNAs (miRNAs), and then function as competing endogenous RNAs (ceRNAs) [9]. The ceRNA-related network could link the functions of lncRNAs, miRNAs, and mRNAs. Dysfunction of these molecules in the network was highly related to the occurrence and development of human diseases, including breast cancer [10].

Although a single gene can function in the study of pathogenesis, detection of individual gene expression can still not promote the overall understanding of human diseases [11]. Recently, the application of biological networks for identifying biomarkers and understanding cancer biology has become increasingly urgent [12]. Networks specific to disease context could help in improving the understanding of the underlying biology from a global perspective [13]. Transcription factors (TFs) are a kind of genes that could function in the regulation of gene expression via binding to their DNA regulatory elements, such as promoters or enhancers [14]. The miRNAs, TFs, and the mRNAs or lncRNAs regulated by them could be integrated for constructing global regulatory networks. More intriguingly, network module centrality analysis provided more information to understand biological problems [15]. However, some regulatory patterns such as lncRNA-TF interactions in breast cancer remained unknown. More important molecular mechanisms underlying breast cancer still need more comprehensive molecular and biological studies.

In the present study, we are working to construct a lncRNA-TF-associated ceRNA network for revealing their potential interaction in breast cancer using bioinformatics tools. This network contained TFs, lncRNAs, and their interactions based on ceRNAs and protein-protein interactions (PPIs). First, we performed a comprehensive analysis of the network and computed important topological features, such as degree and average path length. Hub nodes with the highest degrees in the lncRNA-TF-associated ceRNA network were selected to detect differential expression in different subtypes/tumor stages of breast cancer. Then, closely connected modules were identified from the lncRNA-TF-associated ceRNA network. Survival analysis was performed to evaluate whether the modules had prognosis effects on breast cancer. Furthermore, in order to investigate the binding potential between TFs and lncRNAs, we performed TF motif searching to indicate the promoter and enhancer regions of lncRNAs. In conclusion, our study could help explain the biological processes and molecular mechanism of breast cancer from a global network perspective.

## 2. Materials and Methods

### 2.1. Breast Cancer-Related Datasets

We downloaded breast cancer-related gene expression profile from TCGA (https://xenabrowser.net/datapages/) and converted transcript-level RNA-seq data into lncRNA/protein-coding gene-level RNA-seq data using GENCODE (https://www.gencodegenes.org/human/) [16]. TFs that we obtained before were further mapped to the protein-coding gene-level RNA-seq data. Data preprocessing and log transformation were performed to these RNA-seq data. Finally, breast cancer-related lncRNA/TF RNA-seq expression profiles were obtained. These data involved 1,215 samples with clinical information. All the raw expression data are supported in Supplementary Tables S1 and S2.

### 2.2. Construct a lncRNA-TF-Associated ceRNA Network

Based on ceRNA theory, we comprehensively analyze lncRNA and TF RNA-seq expression profiles of breast cancer and constructed a lncRNA-TF-associated ceRNA network. First, we downloaded all the miRNA-mRNA interactions that were curated from StarBase (http://starbase.sysu.edu.cn/), which contained 386 miRNAs and 13,861 mRNAs (supported in Supplementary Table S3). The miRNA-TF interactions were further extracted by mapping TFs to the mRNAs obtained previously. In addition, we used miRanda tools for identifying significant miRNA-lncRNA interactions by inputting lncRNA and miRNA sequences (default parameters) [16]. Second, we counted the number of the shared miRNAs for each lncRNA-TF pair based on the miRNA-TF interactions and miRNA-lncRNA interactions and indicate the shared miRNAs with statistical significance for all the lncRNA-TF pairs using hypergeometric test. The lncRNA-TF pairs with the threshold of hypergeometric test *p* value < 0.05 were considered statistically significant (Supplementary Table S4).

Third, using breast cancer-related lncRNA and TF-level RNA-seq expression profiles, Pearson correlation coefficients (PCC) were further calculated for those lncRNA-TF pairs with a hypergeometric test *p* value < 0.05. And the lncRNA-TF pairs with PCC > 0.6 were finally considered significant lncRNA-TF pairs (Supplementary Table S5).

In addition, TF-related PPI interactions were extracted from the HPRD database. Then, a breast cancer-related lncRNA-TF ceRNA network was formed by combining all the significant lncRNA-TF pairs and TF-TF PPI pairs (Supplementary Table S6).

### 2.3. Identify Closely Connected Network Modules

We used the Molecular Complex Detection (MCODE) plug-in in Cytoscape to identify closely connected modules from the lncRNA-TF-associated ceRNA network. The MCODE algorithm is based on graph-theoretical analysis, which clusters a given network by topology for finding densely connected regions [17]. The criteria that we used for identifying functional modules were as follows: MCODE scores > 5, degree cutoff = 2, node score cutoff = 0.2, max depth = 100, and *k* − score = *c*2.

### 2.4. Survival Analysis

Our gene expression profile contained 1,215 breast cancer patients with clinical information. Subtype classification is defined from TCGA clinical matrix. Based on these data, the univariate Cox regression was used to identify breast cancer-related prognostic signatures. We accumulated the regression coefficient and the expression values of each gene and computed the risk score of each patient as follows:

(1) $$\begin{array}{c} Risk\;score=\overset{n}{\underset{i=1}{\sum }}r_{i}Exp\left(i\right), \end{array}$$

where *n* is the number of genes in a gene set, *r*_*i*_ is the regression coefficient of gene *i*, and Exp(*i*) is the expression value of gene *i* for a corresponding patient.

We classified breast cancer patients into two groups by using the mean risk score as a cutoff. That is, patients with the risk score greater than the mean value were classified into a high-risk group. Patients with the risk score less than the mean value were classified into a low-risk group. These high-risk group and low-risk group patients were then used to perform Kaplan-Meier survival analysis. Log-rank test with a *p* value < 0.05 was used to generate statistical significance. The raw TCGA clinical matrix is supported in Supplementary Table S7.

### 2.5. TF Motif Searching Analysis

For investigating the binding potential between TFs and lncRNAs, we performed TF motif searching analysis to the promoter and enhancer regions of lncRNAs. Promoters were defined as +/-2 kb from transcription start site (TSS). Enhancers were downloaded from FANTOM5 [18, 19]. FIMO was used to scan promoter and enhancer regions with a *p* value < 1*e*–4 [20].

## 3. Results

### 3.1. Construction of a lncRNA-TF-Associated ceRNA Network

**Figure 1:**
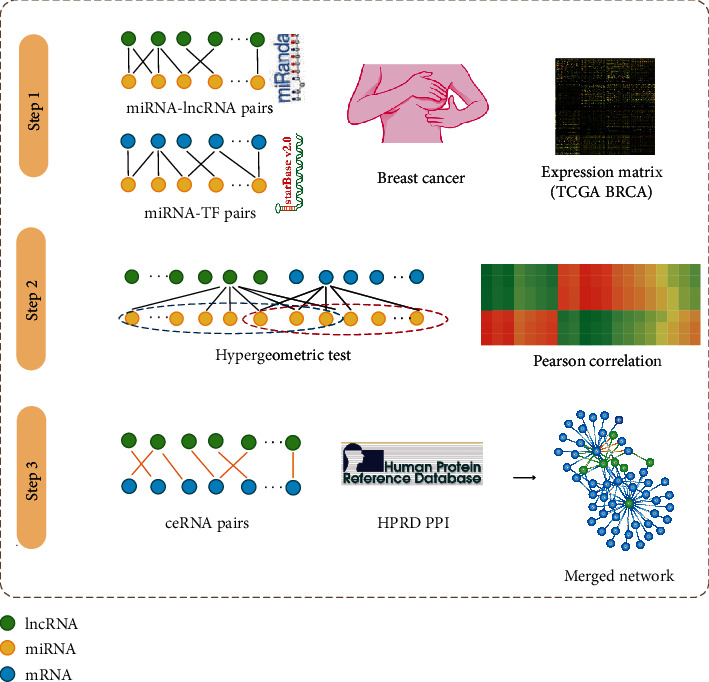


**Figure 2:**
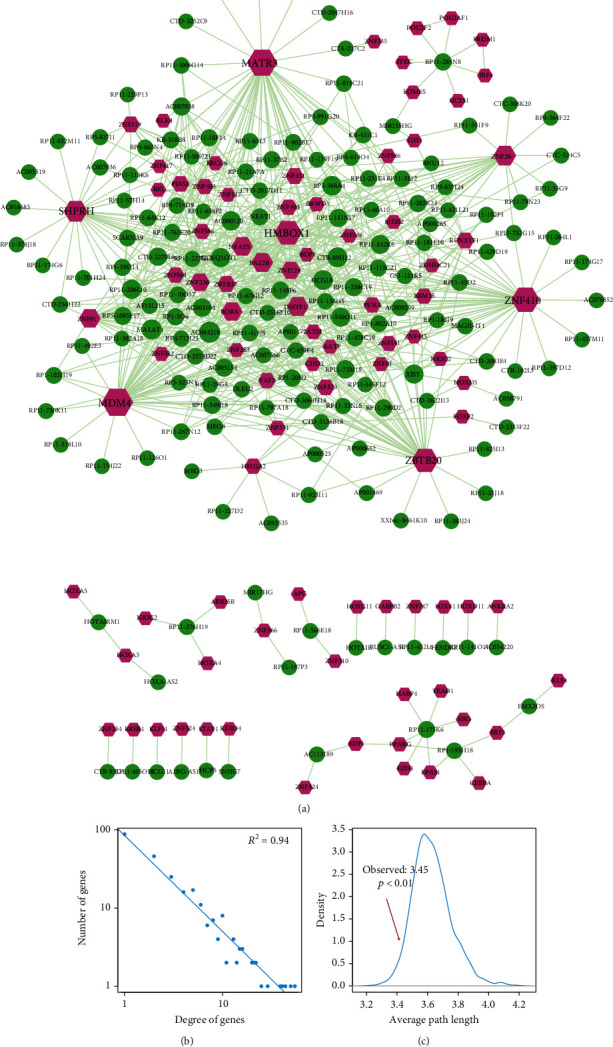


lncRNAs that contained miRNA-response elements could competitively bind miRNAs with mRNAs and then function as ceRNAs to participate in multiple biological processes of complex diseases. In this study, a lncRNA-TF-associated ceRNA network in breast cancer was constructed by combining all significant lncRNA-TF ceRNA pairs and TF-TF PPI pairs (Figure 1, details in methods). This network consisted of 164 lncRNA nodes, 91 TF nodes, and 644 edges (Figure 2(a)). To evaluate the importance of network nodes, we performed topological analyses for the lncRNA-TF-associated ceRNA network (Supplementary Table S8). First, we computed the degree of network nodes and found that all the nodes followed a power law distribution, which indicated that the network had the scale-free property (Figure 2(b), *R*^2^ = 0.94). Next, we calculated the average path length of the lncRNA-TF-associated ceRNA network. Simultaneously, we also chose 1,000 degree-conserved random networks to calculate their average path length and counted the number of average path length in a random network shorter than that in the real network. *p* values were calculated by the number divided by 1,000. The result showed that the average path length of the real network was significantly shorter than that of random networks (Figure 2(c), *p* < 0.01). These results suggested that hub genes of the lncRNA-TF-associated ceRNA network played important roles in the local region of the network.

### 3.2. Detection of Breast Cancer-Related Hub Genes

**Figure 3:**
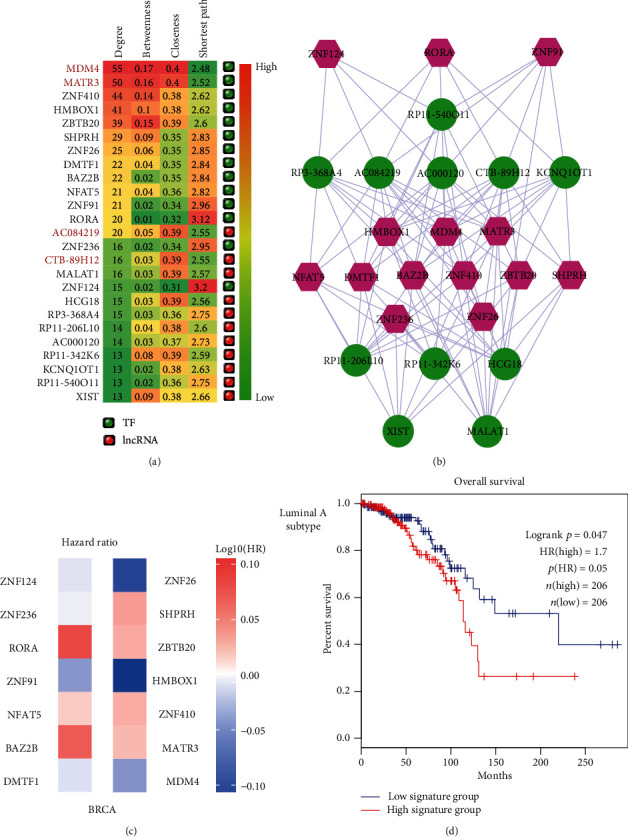


Numerous studies found that genes connected by a large number of other genes (also known as high degree) in biological network tended to play vital roles in pathological processes. These genes with high degree in network were defined as hub genes. Here, we detected breast cancer-related hub genes from the lncRNA-TF ceRNA network. We defined the genes with top 10% node degree as hub genes, including 14 TFs and 11 lncRNAs (Figure 3(a)). Results showed that these hubs not only had high degrees but also had high betweenness, closeness, and low shortest path length, indicating that these genes might maintain the basic biological processes in cancer pathology (Figure 3(a)). We further extracted the hub-hub subnetwork from the lncRNA-TF-associated ceRNA network. As a result, the hub-hub subnetwork was composed of all these hubs and their 103 edges, including the known cancer-related lncRNAs *MALAT1* and *XIST* (Figure 3(b)). Then, we tested the prognosis effects of the 14 hub TFs. Results showed that hazard ratios of these TFs in breast cancer of TCGA BRCA cohorts were not significant (Figure 3(c)). However, in luminal A subtype, these hub TFs showed a strong prognosis effect (Figure 3(d)). These results inspired us to investigate the function of hub genes in subtypes of breast cancer.

**Figure 4:**
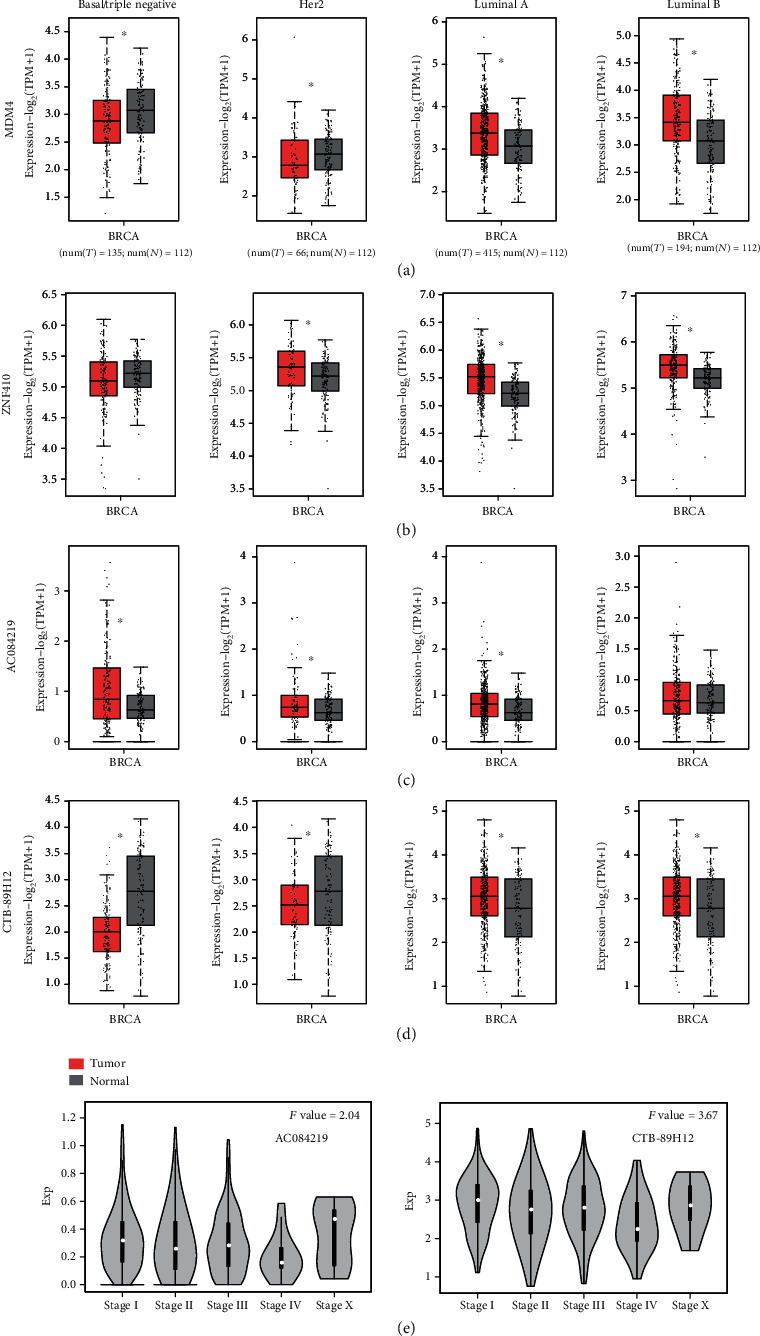


The results mentioned above showed that our lncRNA-TF-associated ceRNA network had the scale-free property, representing a small subset of high-degree nodes (also called hubs) that were connected by the most of other nodes. Thus, we selected 2 TF hubs (*MDM4* and *ZNF410*) and 2 lncRNA hubs (*AC084219* and *CTB-89H12*) with the highest degrees from the lncRNA-TF-associated ceRNA network and detected their expression in various subtypes of breast cancer. The results showed that they could significantly be distinguished between breast cancer samples and normal samples (Figures 4(a)–4(d)) in different subtypes. Actually, *MDM4* has been emerging as an important breast cancer biomarker and oncoprotein [21]. *MDM4* was found highly expressed not only in normal breast epithelial cells but also in most luminal breast cancer [22]. *MDM4* has also been suggested to promote triple-negative breast cancer metastasis [23]. Cancer cells and stromal/immune cells, such as cancer-associated fibroblasts, were the important parts of tumor microenvironment. *ZNF410*, also known as *APA-1*, was a TF that regulated the expression of matrix-remodeling genes during fibroblast senescence [24]. Du et al. have shown the tumor-suppressive role of lncRNA *CTB-89H12* and the expression regulation ability of *PTEN* in prostate cancer [25]. The above studies suggested that hub nodes in global lncRNA-TF network might play important roles in biological processes and molecular functions of breast cancer. We further calculated the expression of the two lncRNAs (*AC084219* and *CTB-89H12*) in different tumor stages and found that they were differentially expressed in the advanced stage of tumor (Figure 4(e)).

### 3.3. Identification of Closely Connected Network Modules

Biological networks are often too large to interpret the biological phenomena accurately. Functional modules of a network may be more useful for reflecting the relevant biological importance. Functional modules have been widely applied to explore the mechanism involved in various biological processes, such as miRNA regulation, disease occurrence, and drug action [26]. We used “MCODE” in the Cytoscape software to identify closely connected network modules from our lncRNA-TF-associated ceRNA network. As a result, two closely connected modules linked to breast cancer were identified.

**Figure 5:**
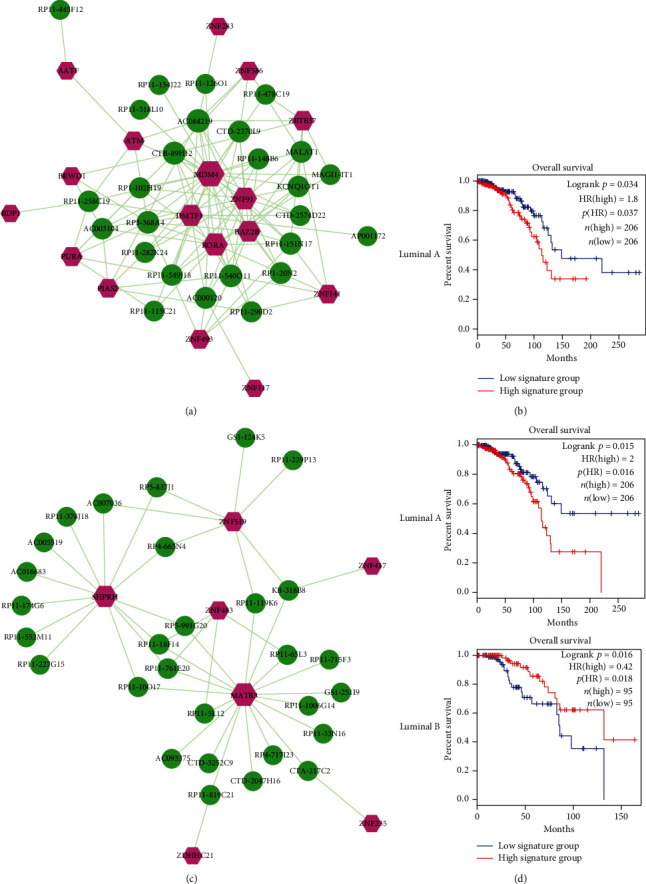


Module 1 was composed of 43 nodes (26 lncRNAs and 17 TFs) and 120 edges (Figure 5(a)). Some lncRNAs and TFs of module 1 have been reported to function in breast cancer. For example, *MDM4* negatively regulated the major tumor suppressor gene *p53* and further modulated stress responses, which had been considered a biomarker that may drive metastasis and progression of breast cancer [27]. The altered expression of *DMTF1* proteins was highly related to the pathophysiology of cancer. In response to oncogenic stresses, *DMTF1* bind to the promoter of *ARF* and governed the *ARF-p53* tumor suppressor pathway activity [28]. LncRNA *PURA* was an evolutionarily conserved cellular protein participating in processes of DNA replication, transcription, and RNA transport, which functioned in human cancer [29]. To evaluate whether module 1 had prognosis effects on luminal A breast cancer, we calculated linear combination of expression values of lncRNAs/TFs in module 1 weighted by the regression coefficient of univariate Cox regression to perform survival analysis. As a result, we significantly classified luminal A breast cancer patients into low-risk group and high-risk group with different clinical outcomes (Figure 5(b)).

Module 2 was composed of 36 nodes (29 lncRNAs and 7 TFs) and 45 edges (Figure 5(c)). We also found that several lncRNAs and TFs in module 2 were highly associated with breast cancer. *MATR3* was a highly conserved nuclear matrix protein, which was widely expressed in various tissues and involved in breast cancer-related biological processes, such as transcription, translation, RNA processing, DNA replication, apoptosis, and chromatin remodeling [30]. Axitinib, a clinically approved drug, could effectively treat cancer patients with aberrant activity of nuclear *β*-catenin. The E3 ubiquitin ligase *SHPRH* was identified as the direct target of axitinib. Treatment with axitinib stabilized SHPRH and increased the ubiquitination and degradation of *β*-catenin [31]. Furthermore, we also calculated linear combination of expression values of lncRNAs/TFs in module 2 weighted by the regression coefficient of univariate Cox regression in order to evaluate whether module 2 had prognosis effects on luminal A and luminal B breast cancer. As shown in Figure 5(d), luminal A and luminal B breast cancer patients were significantly classified into low-risk group and high-risk group with different clinical outcomes, respectively. These results suggested that the integration of lncRNAs and TFs in our functional modules had significant prognosis capability and could be used as prognostic signatures of breast cancer.

### 3.4. Identification of Core lncRNA-TF Crosstalks

**Figure 6:**
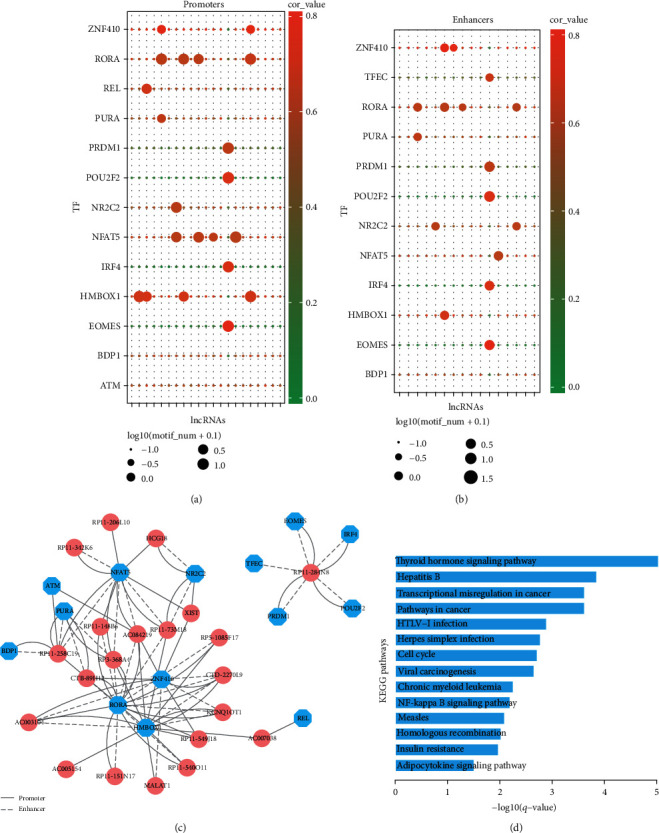


TFs may control the activity of lncRNAs via binding to the DNA regulatory elements of lncRNAs. In this study, we conducted motif searching to the promoter and enhancer regions of lncRNAs for investigating the binding potential between TFs and lncRNAs. The results showed multiple TF binding sites in the promoters and enhancers of lncRNAs, respectively (Figures 6(a) and 6(b)). For example, *NFAT5* has been implicated in cancer cell proliferation and invasion [32]. In this study, *NFAT5* had ceRNA relationships with lncRNAs under the threshold of hypergeometric test *p* value < 0.05 and PCC > 0.6, which were further validated to have multiple motifs binding in the promoters of lncRNAs (Figure 6(a)).

Because hub genes often play more important roles in the biological network, we focused on the motif searching results of top 20% hub lncRNAs in our breast cancer-related lncRNA-TF ceRNA network. Those lncRNA-TF pairs with TFs binding in the promoters and enhancers of top 20% hub lncRNAs were extracted to form a new network (Figure 6(c)). That is, lncRNA nodes and TF nodes in this network had not only significant ceRNA relationships but also strong motif binding. The results implied that TFs might bind to the promoter and enhancer regions of important hub lncRNAs and form “feedback loops” to function in cancer biology. The results of KEGG pathway enrichment showed that TFs of the network were associated with basal functions, such as “Thyroid hormone signaling pathway,” “Hepatitis B,” “Transcriptional misregulation in cancer,” “Pathways in cancer,” and “Cell cycle” (Figure 6(d)). These pathways were all demonstrated to be closely associated with breast cancer [33–35]. For example, breast cancer patients during or after chemotherapy were found to have a remarkable clinical problem of hepatitis B virus [36]. In normal cells, thyroid hormones could regulate the normal physiological processes. However, once signaling pathways became dysregulated, thyroid hormones would induce cancer cell proliferation [37]. Insulin resistance that attenuated biological response to insulin circulation was reported to be associated with a series of pathological conditions and some endocrine tumors, including breast cancer [38]. All these results showed that TFs could crosstalk with lncRNAs via binding to the promoter and enhancer regions of lncRNAs, which were involved in breast cancer-related biological processes and molecular functions.

## 4. Discussion

Breast cancer is accountable for the plurality of cancer deaths among women worldwide. Metastatic breast cancer is even considered an incurable disease with poor prognosis [39]. There is an urgent need to investigate the molecular mechanism and find the significant risk factors for diagnosis and prognosis of breast cancer. The ceRNA regulation may represent a widespread layer of gene regulation which is important for pathogenesis such as breast cancer [40]. Systematically analyzing the lncRNA-related ceRNA network may provide valuable insight into the function of lncRNAs and the molecular mechanism of diseases. Thus, in this study, we constructed a global lncRNA-TF network for revealing their potential interaction in breast cancer using bioinformatics tools. This network was constructed by combining all significant lncRNA-TF ceRNA pairs and TF-TF PPI pairs. First, we made a comprehensive analysis of the network and computed important topological features, such as degree and average path length. We found that all the nodes followed power law distribution and average path length of the real network was substantially shorter than that of random networks. We selected hub nodes with the highest degrees in the global lncRNA-TF network and found that they could significantly distinguish between tumor samples of different subtypes/tumor stages and normal samples. The literature evidences further suggested the importance of hub nodes in the global lncRNA-TF network. Then, two closely connected modules containing some hub genes such as *MDM4*, *DMTF1*, *RORA*, and *MATR3* were identified from the global lncRNA-TF network, which represented significant different clinical outcomes between the breast cancer patients in the low-risk group and high-risk group classified by the survival analysis.

Here, as a point of innovation, we identified some subtype-specific prognosis factors in breast cancer. In Figure 3(c), results showed that TFs have the weak prognosis effects on panbreast cancer. However, combining these factors showed a strong prognosis effect in the luminal A subtype, which indicated that these crucial genes have an important clinical value in luminal A breast cancer. As previously mentioned, *MDM4*, which is a negative regulator of *p53*, not only played crucial roles in regulation of normal breast development but also contributed to the relapsing and metastasis of breast cancer. Intriguingly, *MDM4* was significantly overexpressed in the luminal A subtype of breast cancer [41]. Thus, several anticancer therapeutic strategies such as *SAR405838* [42], *DS-3032b* [43], and *ALRN-6924* [44] were explored with the purpose of restoring the normal activity of p53. As for the *DMTF1* in module 1, Tian et al. found that *DMTF1β*, a major subtype of *DMTFs*, was overexpressed in breast cancer tissues and promotes tumorigenesis in a transgenic mouse model [45]. Niklaus et al. indicated the cisplatin resistance of breast cancer cells is associated with expression of *DMTF1-β* by using SKBR3 (cisplatin sensitive) and MCF7 (cisplatin resistant) breast cancer cell lines in vitro [46]. Maglic et al. demonstrated that overexpression of *DMTF1β* was associated with poor clinical outcomes, by examining the expression of *DMTF1β* in the cancer and adjacent tissue from twenty breast cancer patients, which suggested that *DMTF1β* could be considered a potential diagnostic index for patients with breast cancer [47]. When it comes to *RORA*, viewed as a member of the circadian genes, it could disrupt endogenous homeostasis and thereby promote endocrine tumor development and accelerate progression resulting from the dysfunction of this gene [48]. Taheri et al. found that one functional polymorphism (rs4774388) of *RORA* was associated with breast cancer risk after performing a comparative analysis between the breast cancer patients and the healthy persons in Iran [49]. Besides, Du and Xu [50] observed that *RORA* suppressed the expression of malignant phenotypes in breast cancer cell lines both in vitro and in vivo, which indicated that *RORA* could be considered an ideal potential diagnostic biomarker and therapeutic target of breast cancer. In the module 2, *MATR3*, known as a vital pathogenic gene of amyotrophic lateral sclerosis, is still poorly understood in the process of cancerization [51]. Just a few studies were conducted; for example, Yang et al. performed Western blot and RNA immunoprecipitation assay to find that the lncRNA *SNHG1* was directly interacted with *MATR3* to promote neuroblastoma progression [52]. Nho et al. observed the “Licochalcone H” could suppress cell viability and induce apoptosis in human oral squamous cell lines by suppression of *MATR3* [53]. In short, previous articles indicated that the hub genes involved in the two modules showed a variety of physiological and pathological functions in breast cancer as an integrated interaction network including lncRNAs and TFs, which have significant prognosis capability and could be used as prognostic signatures of breast cancer.

Furthermore, TF motif searching analysis was performed to demonstrated that TFs might bind to the enhancers or promoters of important hub lncRNAs and form “feedback loops” to participate in cancer biology. The enriched pathways were shown to be closely associated with breast cancer; for example, the thyroid hormone signaling pathway ranked as having the highest degree of enrichment. Numerous studies were conducted to study the close relationship between the thyroid hormone and breast cancer. Hercbergs et al. indicated that thyroid hormone promoted the proliferation of the breast cells in vitro and breast cancer cases with hypothyroid function were less likely to be associated with lymph node metastases [54]. Søgaard et al. pointed out that hyperthyroidism was a risk factor for the incidence of breast cancer based on a population-based cohort study [55]. Besides, the NF-kappa B signaling pathway ranked the top 10 signaling pathway in our analysis, indicating a vital role in the regulation of breast cancerization. Liu et al. showed that *lncRNA NKILA* could block the phosphorylation of I*κ*B in vitro and suppress the metastasis of breast cancer by comparison of the different expressions of NKILA between the benign breast tissues and invasive carcinomas [56].

In summary, we provided a comprehensive analysis of breast cancer-related lncRNA-TF ceRNA crosstalk. The results demonstrated that the synergistic, competitive lncRNA-TF pairs played important roles in pathological processes of breast cancer and had strong effect on the prognosis of breast cancer patients. Although our study showed valuable results associated with breast cancer, there were still some limitations. First, we integrated hypergeometric test and PCC computed by gene expression profile to identify significant lncRNA-TF interactions. A stricter measure will decrease false-positive rate and increase accuracy and reliability of our results. Second, we only used FANTOM5 enhancer data to investigate the regulatory loops between TFs and lncRNA enhancers. If we can download the same-sample multiomics data from TCGA, the core lncRNA-TF feedback loops would be more accurate. Third, in this study, we conducted a bioinformatics analysis to identify the crucial factors in breast cancer; results indicated that some genes (TFs or lncRNAs) might play vital roles in the subtype cancers. These results also encouraged us to validate the biological function and mechanism. In further study, we will conduct the biological experiments to investigate these potential factors. In a word, the identified lncRNAs and TFs in the global lncRNA-TF subnetwork and closely connected modules would provide important information for further breast cancer studies and be worth the experimental validations.

## Data Availability

The raw data used to support the findings of this study are available from the Supplementary Materials.

## Supplementary Materials

Supplementary 1 Supplementary Table S1: raw data for processed mRNA expression from TCGA.

Supplementary 2 Supplementary Table S2: raw data for processed lnc expression from TCGA.

Supplementary 3 Supplementary Table S3: raw data for miRNA-mRNA interactions from StarBase.

Supplementary 4 Supplementary Table S4: raw data for hypergeometric results between lncRNAs and TFs.

Supplementary 5 Supplementary Table S5: raw data for PCC results between lncRNAs and TFs.

Supplementary 6 Supplementary Table S6: raw data for network interactions in Figure 2(a).

Supplementary 7 Supplementary Table S7: raw data for clinical information of TCGA breast cancer.

Supplementary 8 Supplementary Table S8: topology feature of genes of the lncRNA-TF-associated ceRNA network.
